# Acute-on-Chronic Liver Failure Triggered by Cutaneous Loxoscelism

**DOI:** 10.7759/cureus.46540

**Published:** 2023-10-05

**Authors:** Francisco Javier Alvarez-Rubio, Arturo Robles-Tenorio, Víctor Manuel Tarango-Martínez

**Affiliations:** 1 Dermatology, Instituto Dermatológico de Jalisco Dr. José Barba Rubio, Guadalajara, MEX

**Keywords:** tropical medicine, recluse spiders, spider bites, cutaneous loxoscelism, acute-on-chronic liver failure

## Abstract

Acute-on-chronic liver failure is a syndrome associated with a high short-term mortality rate. Severe systemic inflammation and single- and multiple-organ failure are a hallmark of this syndrome, with pro-inflammatory precipitating events occurring in the liver or extrahepatic regions. We report a case of a 69-year-old man with a previous diagnosis of alcohol-induced liver cirrhosis who presented with a poorly defined, erythematous-purplish, and edematous plaque with multiple hemorrhagic blisters over the left leg, one day after receiving a spider bite. During the following hours, the skin lesion progressed, and the patient developed hepatic encephalopathy, respiratory failure, and arterial hypotension, requiring the administration of vasopressors; blood analysis revealed hypercreatininemia, an elevated international normalized ratio (INR) value, and hyperbilirubinemia. The patient was diagnosed with acute-on-chronic liver failure caused by cutaneous loxoscelism. There was no hemolytic anemia, rhabdomyolysis, or disseminated intravascular coagulation in the patient, thus excluding the possibility of visceral loxoscelism.

## Introduction

Acute decompensation (AD) of cirrhosis and acute-on-chronic liver failure (ACLF) can be considered opposites in severity, with the latter being more severe than a typical AD. The ACLF presents with higher systemic inflammation and microvascular and mitochondrial alterations, which cause multiple organic dysfunctions, resulting in superior overall mortality [1]. The importance of its rapid recognition is the implementation of multiple therapeutic measures. The 28-day and 90-day mortality rates are higher among patients with ACLF than those with traditional AD cirrhosis (34% and 51% vs. 5% and 14%, respectively) [2]. Patients with ACLF comprise 5% of all hospitalizations for cirrhosis [3], and those discharged following admission have a 30-day readmission rate of approximately 30% [4]. There are hepatic and extrahepatic causes of ACLF. Of the identifiable etiologies, bacterial infections are the most frequent (up to 33%) [2]. Between 40% and 50% of patients have an unrecognized precipitating event that culminates in ACLF [4].

Spider bites of the Loxosceles genus can cause mild-to-severe local involvement, with possible systemic alterations, due to its potent venom that contains enzymes with cytotoxic and hemolytic properties such as hyaluronidase, esterases, and lipases. The most prominent of the enzymes is phospholipase D, which activates complement, attracts polymorphonuclear cells, induces platelet aggregation, and stimulates the release of cytokines and chemokines such as interleukin-8 [5].

We report the case of a patient with severe cutaneous loxoscelism who developed multiple organ dysfunction shortly after hospitalization. Collaboration between different disciplines was necessary for a correct diagnosis and appropriate treatment. Spider venom from Loxosceles may trigger ACLF in patients with chronic liver disease and cirrhosis, either through a local effect that releases cytokines and chemokines that can cause a systemic pro-inflammatory state or a direct hepatotoxic effect.

## Case presentation

**Figure 1:**
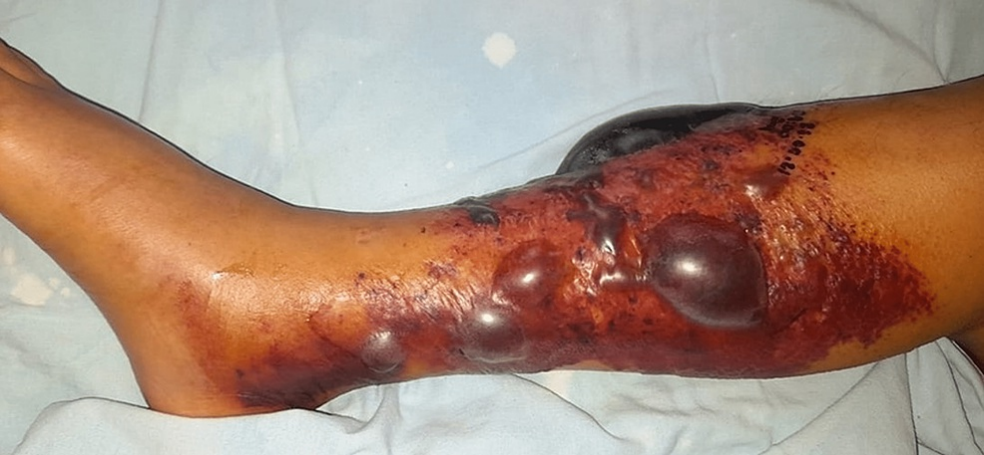
An erythematous swelling with multiple hemorrhagic bullae on the left leg.

A 69-year-old man presented to the emergency room with pain, swelling, discoloration, and hemorrhagic blisters on his left leg, growing in size one day after receiving a probable spider bite while gardening. The patient had a history of binge drinking for 30 years but abstained for the past three years. Type 2 diabetes mellitus was under control with metformin and dapagliflozin, systemic arterial hypertension was being treated with losartan, and the patient also had alcohol-induced liver cirrhosis classified as Child-Pugh B. His last hospital admission was three years before for bleeding in the upper digestive tract due to esophageal varices that required endoscopic variceal ligation and endoscopic sclerotherapy. The patient was started on propranolol 20 mg twice daily after discharge. Physical examination showed a poorly defined, erythematous-purplish, and edematous plaque of 23 cm x 15 cm with multiple hemorrhagic blisters over the left leg (Figure 1). 

Cellulitis was clinically diagnosed. During the next few hours, he developed progressive swelling of the cutaneous lesion, hepatic encephalopathy (West Heaven III), respiratory failure (PaO2/FIO2 of 141), and arterial hypotension (mean arterial pressure of 50 mmHg), requiring a vasopressor.

Blood analysis showed mild leukocytosis (10 x 10^3 ^µL^-1^), elevated INR values (5.3), hypercreatininemia (3.94 mg/dL), mild elevation of lactate dehydrogenase (600 mg/dL), and indirect (7.5 mg/dL) and total bilirubin (10 mg/dL). Blood gas showed metabolic acidosis. No occurrence of hemolytic anemia, rhabdomyolysis, or disseminated intravascular coagulation. Blood cultures taken before antimicrobial treatment with meropenem did not show the growth of microbes after seven days. CT scans showed increased density in superficial soft tissues but no gas, fluid collections, or inflammation under the muscle fascia.

Given the clinical characteristics presented, cellulitis was diagnosed, which led to antibiotic treatment. However, there was a strong argument against this diagnosis since hemorrhagic blisters are not typical of common soft tissue infections. Furthermore, broad-spectrum antibiotics produced no improvement. Therefore, several other conditions were considered, including necrotizing fasciitis. Consequently, amputation of the patient's lower limb was deemed necessary to ensure his survival; however, there was a high possibility of death in a surgical act due to the precarious conditions of the patient. There was a discussion regarding the possibility of cutaneous-visceral loxoscelism. Several factors supported the diagnosis, including the patient's history of gardening, the possibility of a spider bite, and the appearance of a bullous lesion that rapidly grew and was accompanied by intense burning and pain. In opposition to this hypothesis, there was no anemia and no evidence of hemolysis or rhabdomyolysis, which are characteristics of loxoscelism with visceral involvement. Considering the clinical characteristics, laboratory results, and imaging studies, the patient was diagnosed with ACLF triggered by probable cutaneous loxoscelism. The Chronic Liver Failure-Sequential Organ Failure Assessment (CLIF-SOFA) score was calculated (16 points), and the patient received an ACLF-Grade 3, with a 93% probability of dying at one month.

Vancomycin and meropenem were employed as broad-spectrum antibiotics to treat the possible diagnosis of necrotizing fasciitis. The patient received several treatments, including intravenous albumin for acute kidney injury, oral lactulose solution for hepatic encephalopathy, fluids, and vasopressors for hemodynamic support, and glucocorticoid therapy. The administration of Loxoceles antivenom was not possible. After three days of hospitalization, the patient presented spontaneous rupture of the hemorrhagic blisters and proceeded to perform dressings and debridement. After six days, the patient recovered and improved neurologically, and vasopressors and antibiotics were discontinued without further complications.

## Discussion

Patients with ACLF have a poor prognosis, regardless of the definition used to diagnose it. According to the European Association for the Study of the Liver-Chronic Liver Failure (EASL-CLIF) Consortium, ACLF refers to cirrhosis patients who experience AD, organ failure (intra- or extrahepatic), and a high short-term mortality rate [6]. A modified sequential Organ Failure Assessment score (the EASL-CLIF Consortium organ failure scoring system) assesses liver, kidney, brain, circulatory system, and coagulation failure [2].

According to the Asian Pacific Association for the Study of the Liver (APSL), ACLF can be diagnosed without extrahepatic organ failure, and the North American Consortium for the Study of End-Stage Liver Disease (NACSEL) proposes that for the diagnosis of ACLF, there must be at least two serious extrahepatic organ failures [7]. Even though ACLF can have a high mortality rate, reversibility is a hallmark of *acute over chronic* as opposed to end-stage conditions.

ACLF is poorly understood pathophysiologically; however, inflammation resulting from an intra- or extrahepatic process, such as infection, is suggested to play a role [7].

The precipitants of ACLF can be hepatic or extrahepatic. The most common triggers are infectious ones, such as spontaneous bacterial peritonitis, urinary tract infections, skin and soft tissue infections, and respiratory infections. Patients with ACLF who suffer from an infectious or unidentified disease should begin antibiotic therapy empirically. Noninfectious precipitating factors include hepatitis associated with alcohol, drugs, surgical and nonsurgical procedures, or other pro-inflammatory processes [8].

Despite a comprehensive search of the indexed literature, we did not identify any published case of acutely decompensated cirrhosis or ACLF caused by a Loxoceles spider bite. However, spider venom has characteristic local and systemic effects. Loxoceles venom may trigger ACLF in patients with chronic liver disease and cirrhosis, either through a local effect that releases cytokines and chemokines that can cause a systemic pro-inflammatory state or a direct hepatotoxic effect.

Loxosceles are tiny spiders, measuring between 1 and 5 cm in length. They are sexually dimorphic, with females being more prominent than males. A unique characteristic of the genus is the presence of six eyes arranged in three pairs. This genus is commonly known in North America as recluse spiders, brown recluse spiders, or violin spiders due to the violin-shaped dorsal surface of the cephalothorax of the adults [9].

Most cases of human poisoning are caused by five species (Loxosceles gaucho, Loxosceles rufescens, Loxosceles laeta, Loxosceles intermedia, and Loxosceles recluse) [9]. Loxosceles venom is associated with dermonecrosis and, to a lesser extent, systemic toxicity (intravascular hemolysis, thrombocytopenia, disseminated intravascular coagulation, and acute renal failure) [10].

Loxosceles bites cause mild itching, and the clinical signs and symptoms appear several hours later. As a result, the bite appears barely noticeable, and the spider is rarely caught (10% of cases) at the time of the bite. Loxoscelism is usually diagnosed presumptively at the time of hospitalization, approximately 12 to 24 hours after the bite when the skin has been severely damaged [10]. Based on clinical and epidemiological data, loxoscelism can be classified as follows [11]:

• Assumption: The spider is not known to be in the area, and the skin lesion is atypical.

• Presumptive: The spider is known to be in the area, the lesion is compatible, and there is a typical clinical course.

• Probable: The spider is in the area, the patient may have felt the bite or seen a spider, the lesion is typical, and there is a typical clinical course.

• Documented: The spider is found after the bite, a qualified person has identified it, the lesion is typical, and there is a typical clinical course.

The clinical classification of loxoscelism is either cutaneous or viscerocutaneous. Edematous erythema may appear two to six hours following the spider bite. An irregular macule containing purple and pale areas, sometimes indurated and surrounded by erythema (red, white, and blue signs), may develop; in severe cases, serous or hemorrhagic blisters may appear; gravitational extension is a hallmark. Around seven to 10 days after the poisoning, 20% of cases progress to a dry, necrotic eschar. A severe injury is more likely to occur on the thighs, buttocks, and abdomen, which contain a greater concentration of fatty tissue. Secondary infections are uncommon [9].

Patients may experience malaise, nausea, vomiting, fever, and myalgias; however, the most severe complications are seen in viscerocutaneous loxoscelism, which includes hemolytic anemia, hematuria, hemoglobinuria, rhabdomyolysis, and disseminated intravascular coagulation [12]. Oliguria and dark urine, which can suggest extensive intravascular hemolysis or rhabdomyolysis, can result in acute renal failure, the primary cause of death associated with loxoscelism [13].

Numerous treatments are available for loxoscelism, such as antivenom, dapsone, antihistamines, analgesics, and corticosteroids, but none is universally accepted. Two types of antivenoms are available for treating loxoscelism (horse-derived F(ab′)2 antivenoms or complete IgG antivenoms), both administered intravenously; all patients affected by viscerocutaneous loxoscelism require antivenom [14]. Other treatments have been used, including surgical excision, hyperbaric oxygen therapy, and negative pressure wound therapy (vacuum-assisted closure) [9].

The venom comprises various components, including phospholipases D, astacin-like metalloproteases, and low-molecular-mass insecticidal peptides (ICK peptides), which comprise most of the transcripts encoding toxins (95%), hyaluronidase, serine proteases, serine protease inhibitors, venom allergens, and a member of the translationally controlled tumor protein (TCTP) family [9]. Phospholipase D is a pro-inflammatory protein that stimulates complement activation through the classical and alternative pathways. Moreover, phospholipase D, directly and indirectly, promotes the production of pro-inflammatory cytokines, such as interleukin-6, interleukin-10, interleukin-8, granulocyte-macrophage colony-stimulating factor, and monocyte chemoattractant protein-1 [15].

In the study by de Oliveira Christoff et al. [16], Loxoceles venom was detected in hepatic tissue by immunofluorescence assays, associated with both morphological and functional damage. The pathology found inflammatory infiltration of mononuclear and polymorphonuclear cells, steatosis, microabscesses, ballooning, and hepatocyte cell death. Initially, the changes are more pronounced at six hours, and then they begin to decrease at 12 hours. Lesions caused by Loxosceles venom resemble those caused by acute viral hepatitis, which has been associated with ACLF. The acute lesions caused by Loxosceles venom were temporary; they did not show evidence of fibrosis, which is characteristic of chronic liver disease. Our patient presented a progressive recovery in a couple of days, which indicates a reversibility of the hepatic exacerbation, data consistent with the reversibility of the changes shown by the murine model of Loxosceles venom [15].

## Conclusions

Based on the solid chronological correlation between the spider bite and the absence of another ACLF precipitant, venom is likely to have been the cause of this exacerbation. We hypothesize that Loxosceles venom can cause liver exacerbation in patients with cirrhosis as it exerts a local and systemic pro-inflammatory effect and hepatotoxicity. Despite a comprehensive search of the indexed literature, we did not identify any published cases of ACLF caused by cutaneous loxoscelism. The importance of reporting this case lies in the potential of the venom to produce a severe systemic inflammatory state capable of developing a hepatotoxic effect with subsequent organ failure.
